# Supplementary material for: Association of lithocholic acid with skeletal muscle hypertrophy through TGR5-IGF-1 and skeletal muscle mass in cultured mouse myotubes, chronic liver disease rats and humans
Source: eLife. 2022 Oct 7;11:e80638. doi: 10.7554/eLife.80638 (PMC9545520; doi:10.7554/eLife.80638)

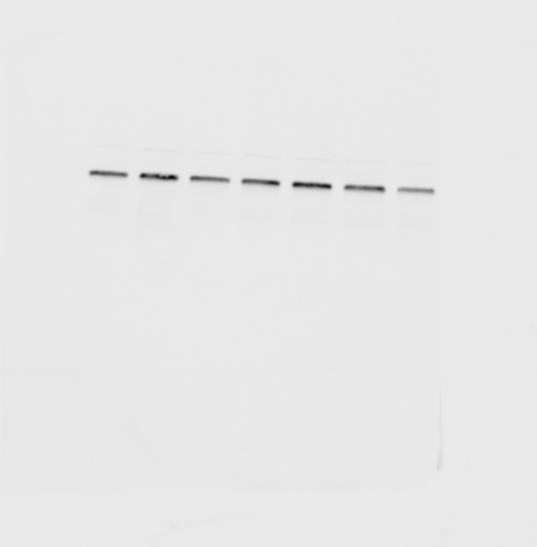

Supplement: Figure 5—source data 2. [file elife-80638-fig5-data2.pdf]
