# Supplementary material for: Association of lithocholic acid with skeletal muscle hypertrophy through TGR5-IGF-1 and skeletal muscle mass in cultured mouse myotubes, chronic liver disease rats and humans
Source: eLife. 2022 Oct 7;11:e80638. doi: 10.7554/eLife.80638 (PMC9545520; doi:10.7554/eLife.80638)
Supplement: Supplementary file 1. [file elife-80638-supp1.docx]

**Supplementary File 1. Forward and reverse qPCR primers used for amplification.**

Gene Forward Reverse

Rat

*MuRF1* 5’- ACACATAGCAGAGGCCTTGAG -3’ 5’- CCTCCTCCTCTTCAGTAAACTCC -3’

*MafBx* 5’- CCATCAGGAGAAGTGGATCTATGTT -3’ 5’- GCTTCCCCCAAAGTGCAGTA -3’

*Ubiquitin* 5’- GATCCAGGACAAGGAGGGC -3’ 5’- CATCTTCCAGCTGCTTGCCT-3’

*E214KDa* 5’- TCCTGCAGAACCGATGGAG -3’ 5’- CGGCTCATCCAACAGAGACTG -3’

*FOXO1* 5’- TCAGGCTAGGAGTTAGTGAGCA -3’ 5’- GGGGTGAAGGGCATCTTT -3’

Mouse

*Gpbar1* 5’- CCTGGAACTCTGTTATCGCTCA -3’ 5’- GCACTCGTAGACACCTTTGGG -3’

*Igf1* 5’- GTGAGCCAAAGACACACCCA -3’ 5’- ACCTCTGATTTTCCGAGTTGC -3’

MuRF1; muscle RING finger 1, MafBx; muscle atrophy F-box protein, FOXO1; forkhead box O1, Gpbar1; G-protein-coupled bile acid receptor 1, Igf; insulin growth factor.
